# The Pathology and Diagnosis of Acute Bright’s Disease

**Published:** 1873-09

**Authors:** George Johnson

**Affiliations:** Physician to King’s College Hospital


### On the Pathology and Diagnosis of Acute Bright's Disease.

By Dr. George Johnson, F.R.S., Physician to King's College Hospital.

While acute Bright's disease is usually associated with a more or less copious epithelial desquamation, there may be no desquamation of renal epethelium, and either no tube-casts or only small hyaline casts in the urine; while in other cases, either with or without epithelial casts, there may be casts crowded with small exudation-cells. The appearances which I have described are sharply defined in some cases, while in others they gradually merge into each other. Epethelial casts and desquamation may be abundant or entirely absent, or present in moderate amount. The casts with exudation cells may be numerous and unassociated with epithelial casts, or the two forms of tube-casts may be combined with and replace each other in variable proportions. It is certainly interesting, and, I think, of some practical importance, to note these different appearances in the urine.

In all the cases of acute Bright's disease to which I have hitherto referrred, although the microscopical appearances in the urine are various, the general symptoms and the physical and chemical characters of the secretion are alike, and in particular the presence of a large amount of albumen is a constant phenomenon. Now, I have to tell you that we sometimes, though rarely, meet with cases of acute general dropsy in which the urine, although scanty, contains not a trace of albumen. In the great majority of cases acute Bright's disease and acute albuminuria are synonymous terms; but in these few exeptional cases the latter term is inapplicable, for the urine is not albuminous. Dr. Blackall described two cases of acute general dropsy after scarlet fever, in which the urine was not coagulable either by heat or by nitric, or, as he calls it, "nitrous" acid (*op. cit.*, p. 12 to 21). Dr. Roberts gives the history of two cases after scarlet fever, both fatal—one acute, the other chronic (On Urinary and Renal Diseases pp. 24 and 400.) Dr. Basham has reported the case of an adult in whom general dropsy followed exposure to wet and cold. He recovered (Lancet, August 1867). And I have notes of four cases that have come under my own observation. Three of these cases recovered, and the fourth was improving when he was lost sight of. In two of my cases the dropsy followed scarlet fever, and in the other two it was, probably a result of exposure to cold. In two of the cases neither albumen nor tube-casts could be discovered throughout ; in one a trace of albumen was found on one occasion; and in the fourth, after general dropsy had existed for six weeks without albumen or tube-casts a trace of albumen and some hyaline casts appeared.

Now what is the explanation of these rare, remarkable, and exceptional cases? I have neither seen nor heard of any satisfactory explanation of them, and I am not prepared to give you one; but I venture upon one or two suggestions and queries. There is reason to believe that suppressed action of the skin is a powerful concurring cause of the dropsy which is associated with albuminuria; and this, perhaps, is the explanation of the frequent association of dropsy with the renal disease which results from scarlet fever or from exposure to cold and wet. In both these classes of cases the functions of the skin must obviously be more or less impaired—in the one by the specific inflammation, and in the other by cold; whereas diphtheritic albuminuria, without implication of the skin in the morbid process, rarely, if ever, gives rise to general dropsy. Then the question arises, Is it possible that suppression of the cutaneous secretion may alone cause acute general dropsy without the implication of the kidneys? May acute general dropsy result from a metastasis of the perspiration from the skin to the areolar tissue and the serous membranes? And may the scanty secretion of urine in these exceptional cases be a result of the morbid transfer of water to the tissues where the dropsical effusion takes place, as, by a reversed action, the perspiration is checked and the skin of a diabetic patient rendered dry by the copious flux of liquid through the kidneys? I am not prepared to answer these questions. In most cases of acute dropsy without albuminuria the urine has been scanty and high colored. In one of Dr. Roberts's cases the urine was scanty almost to suppression, only two drachms having been voided in twenty-four hours; "it contained casts, but not a trace of albumen." The form of tube-casts is not mentioned. The total quantity of urine voided during the last seven days of life amounted to between six and seven ounces. No autopsy was permitted. In the second case the urine contained neither albumen nor casts, but it was scanty and high colored; and, death having occurred after an illness of five months, "the kidneys were found to be good examples of the smooth white Bright's kidney." In this case it would seem that, although there was no albuminuria, there was some structural change in the kidneys. Dr. Wilks has published in the sixth volume of the Pathological Transactions a remarkable case of general dropsy, with a peculiar form of renal disease, but without albuminuria, in a woman aged 35. The urine passed amounted to about twelve ounces in the day, of specific gravity 1012, and not albuminous. A few days before death the urine became less in quantity, and for the last four days none was obtained. The kidneys were pale and large, their combined weight being seventeen ounces. The cortical portion was seen by the naked eye to be scattered over with small round dots like grains of sand. On a microscopic examination these were found to be the

Malpighian bodies, the capillaries of which were covered over with mulberry-like masses of oil-globules, while the tubes were healthy.

It may hereafter happen to some of you to have the opportunity of throwing additional light upon the pathalogy of these rare and exceptional cases of general dropsy not dependent on heart-disease and unassociated with albuminuria.

We have seen that the chief varieties and modifications of acute Bright's disease with albuminuria are the following:—1, with epithelial desquamation (desquamative nephritis); 2, without desquamation, either with or without small hyaline casts; 3, with exudation-cell casts, either with or without epithelial casts and desquamation. Lastly, we have, as an entirely distinct class of cases, rare, exceptional, and obscure in their pathology, acute Bright's disease, or at any rate acute general and febrile dropsy, without albuminuria.

*Changes in the Blood.*—The effect of acute Bright's disease is not only to cause an admixture of blood-constituents with the urine, but also to bring about a large accumulation of urinary materials in the blood. While the urine is usually more or less bloody, the blood becomes in a greater or less degree urinous. Dr. Christison was the first to announce the fact that the blood in these cases contains a large amount of urea and that urea is found in the dropsical and inflammatory effusions (Edinburgh Medical and Surgical Journal, October 1829). Not only is the blood altered by an accumulation of urinary materials, but also by a loss of its own normal constituents. The density of the serum is reduced from 1030 or 1031 to 1022 or even 1020. The loss of density is greatest when the urine has been most albuminous; and it is probably explained by the escape of serum through the kidneys. The hæmoglobin or coloring matter also diminishes rapidly, the normal proportion being 1,335 in 10,000. Dr. Christison found it reduced, after a few weeks' illness, as low as 955 in one case, in another to 564; and in a young man ill for three months and a half subsequent to scarlet fever, who had never been bled before, it was only 427. At the commencement of the disease the loss of coloring matter is less rapid than the extreme pallor of the patient would seem to indicate; and it is probable that the blanched appearance of the skin is partly occasioned by the quantity of water in the subcutaneous tissue.

*Etiology.*—Acute Bright's disease may occur at all ages from infancy to extreme old age. The two most frequent causes of acute Bright's disease with dropsy are exposure to wet and cold and scarlet fever. Either of these causes is alone sufficient to excite the disease; but their combined action—exposure to cold during the progress of scarlet fever—is a most powerful determining cause of the malady. Diphtheria is a frequent cause of albuminuria; but, as I have before said, diphtheritic albuminuria is rarely associated with dropsy. Amongst the less frequent causes of acute albuminuria are measles, erysipelas, pyæmia, the absorption of poisonous materials from the uterus after parturition, rheumatic fever, the malarious poison, typhus and typhoid fever, cholera, and, lastly, excessive eating and drinking, more especially when combined with dyspepsia. In the majority of cases acute albuminuria, resulting from other causes than scarlet fever and exposure to cold, is unassociated with dropsy, and its history belongs to that of the disease with which it is associated as a complication. We shall find hereafter that albuminuria resulting from one or other of the various causes here referred to sometimes leads to a chronic and incurable degeneration of the kidney. Excess of alcohol is a more frequent cause of chronic than of acute Bright's disease. A remarkable case of transient alcoholic albuminuria occurred when Dr. Baxter was house-physician to our hospital. A man between twenty and thirty years of age was brought in one night by the police. He was unconscious, and breathing stertorously. He was believed to be drunk, and a large quantity of vinous liquor was pumped out of his stomach. The unconsciousness remaining, uræmia was suspected, and some urine drawn off with the catheter was "loaded with albumen." He was then put into bed, cupped over the loins, and a purgative given. When Dr. Baxter visited the ward in the morning he found the man sitting up and clamoring for his discharge. He said that he had been very drunk over night, but now he had nothing the matter with him. He passed some urine, which was found of normal color and specific gravity, and without a trace of albumen. He then left the hospital in triumph. The temporary albuminuria was the result of renal congestion while the excess of alcohol was being excreted by the kidneys.

*Diagnosis.*—In most cases of acute Bright's disease the symptoms are so obvious, that the disease can scarcely be overlooked or mistaken for any other. The only case in which there is a possibility of acute albuminuria being unrecognized are those in which it is unassociated with dropsy. But, the existence of albuminuria being discovered, it is not always easy to determine whether this is the result of a recent acute attack, or of a chronic degeneration of the kidney. We shall be in a better position to discuss this important practical question after we have studied the various forms of chronic Bright's disease. Meanwhile, however, I may tell you that, as a rule, high colored, smoky and blood-tinged urine, of high specific gravity, is an indication of a recent acute attack; and equally so is a copious sediment composed of epithelial and blood-casts, or of exudation-cell casts, alone or mixed with epithelial casts. The appearance of oily casts and cells, in combination with numerous epithelial casts, does not materially affect the diagnosis. On the other hand, urine of low specific gravity and very pale in color, yet highly albuminous, is usually evidence of chronic disease; and this evidence is strengthened by the appearance of numerous oily casts and cells unassociated with epithelial or exudation-cell casts. Large hyaline casts in *pale* highly albuminous urine point to disease not only chronic, but in an advanced stage. We shall return to the subject, and discuss it more fully in a future lecture.

*Prognosis.*—Acute Bright's disease has a tendency to terminate in complete recovery. It is essentially a curable disease, as much so as acute bronchitis or acute pneumonia. The earlier the patient comes under treatment the better is his prospect of recovery; and, on the other hand, the longer the symptoms have continued without signs of amendment, the more grave does the prognosis become. The prognosis is, on the whole, more favorable in the young and middle-aged than in those more advanced in years; but the disease may prove mild and tractable even in very aged persons. For obvious reasons, the prospect of recovery is better in the case of those who can avoid exposure to cold and other injurious influences, than when the patients' circumstances are less favourable.

In favourable cases a copious secretion of urine, of comparatively low specific gravity and of paler colour, with a diminishing amount of albumen and decrease of dropsy, are amongst the earliest signs of amendment. Albuminuria is usually the last symptom to disappear. The time of its disappearance varies, in different cases of recovery, from a few days to many months. If the urine continue albuminous for more than six months, it becomes more and more doubtful whether it will ever cease to be so; but I have seen cases of complete recovery after albuminuria had continued for one, two, and even three years. So long as the urine continues albuminous, in however slight a degree, although the dropsy and all other general symptoms may have passed away, recovery must be considered incomplete. Acute Bright's disease, although, as a rule, a curable, is not unfrequently a fatal disease. There are some symptoms and complications which indicate a case of more than ordinary peril; such as a very scanty secretion of highly albuminous urine; frequent and distressing vomiting; great anasarca, with a tendency to erysipelatous inflammation of the skin; dropsical effusion within the chest, either in the pleura or the pericardium, or both, with urgent dyspnœa; inflammatlon of the lung, or pleura, or pericardium, or endocardium; severe and persistent headache, which is apt to be followed by convulsions and by coma, with a brown and dry tongue. All these are symptoms of grave, though not always of fatal import. When the renal disease is acute, and therefore essentially curable, recovery sometimes occurs after the most formidable symptoms of uræmic poisoning have been present.

A consideration of the exciting causes of the renal disease forms an element in the prognostic indications. When Bright's disease results from some inherent constitutional defect, without obvious exciting cause, it is generally more intractable than when it is directly due to exposure to cold or to the influence of some specific blood-poison, as, for instance, that of scarlet fever or erysipelas. To all general rules of this kind there are exceptions, and each case requires a separate and careful study.

Let me impress upon you one point of practical importance. Before you pronounce a patient to be entirely free from his malady, be careful to test the urine, not only after rest and fasting—*i. e.*, in the morning before breakfast—but after food and exercise. Albuminous urine is usually more copiously so after food and exercise; and you will sometimes find that, while the urine before breakfast is quite free from albumen, that which is secreted after a meal is decidedly and even copiously albuminous. In some cases exercise has even more influence than food in exciting renal congestion and albuminuria.

An attack of acute Bright's disease confers no immunity from future attacks; on the contrary, the disease may occur more than once in the same subject, a result of either inexplicable predisposition, or of a liability resulting from a first attack. I think my experience warrants the statement that when acute albuminuria has resulted from some non-specific cause, such as exposure to cold and wet, or excessive eating and drinking, it is more likely to recur than when it has been excited by a specific morbid poison, such as that of scarlet fever, which, as a rule, does not occur a second time in the same individual; but I have known patients so unfortunate as to have two attacks of scarlet fever, and each attack complicated with albuminuria.—*British Medical Journal.—Braithwaite's Retrospect.*

